# Lineage-Specific CagA Binding Mechanics and Microenvironmental Rewiring in East Asian Gastric Carcinogenesis

**DOI:** 10.3390/molecules31173118

**Published:** 2026-09-06

**Authors:** Hongbo Xie, Denan Zhang, Lei Liu, Qing Jin, Xiujie Chen

**Affiliations:** Department of Pharmacogenomics, College of Bioinformatics Science and Technology, Harbin Medical University, Harbin 150086, China; zhangdenan@ems.hrbmu.edu.cn (D.Z.); liulei@ems.hrbmu.edu.cn (L.L.); jinqing@hrbmu.edu.cn (Q.J.)

**Keywords:** *Helicobacter pylori*, molecular dynamics, cytotoxin-associated gene A, gastric cancer

## Abstract

Chronic infection with *Helicobacter pylori* (*H. pylori*) is a major environmental risk factor for gastric carcinogenesis. Malignancy is largely driven by variations within the virulence factor CagA, with East Asian lineages exhibiting higher oncogenic potential than Western ones. However, how these variants modulate cellular crosstalk remains poorly understood. We integrated molecular dynamics (MD) simulations with single-cell transcriptomics across progressive disease stages, including chronic atrophic gastritis, intestinal metaplasia, and gastric cancer. Local niche remodeling was evaluated via cell–cell communication profiling among epithelial, stromal, and immune circuits, while simulations of MARK2 kinase bound to distinct CagA lineages determined binding affinities. Single-cell analysis revealed that *H. pylori* toxicity progressively dampens epithelial–stromal crosstalk, marked by severe epithelial polarity aberrations that disrupt neuroendocrine-like secretory and synaptic pathways during malignant transformation. Mechanistically, MD simulations and MM/GBSA calculations demonstrated that East Asian CagA lineages exhibit higher binding affinity toward host MARK2 than Western lineages. Specific East Asian amino acid substitutions dramatically tighten the protein interface, driving stronger signaling perturbations. This study bridges atomistic structural virulence with microenvironmental shifting, establishing geographic CagA toxicity divergence as a critical determinant for pathogen-driven gastric cancer risk.

## 1. Introduction

*Helicobacter pylori* (*H. pylori*) infection is one of the most prevalent chronic bacterial infections worldwide, affecting approximately 50% of the global population [1,2]. It is a well-established etiological factor in a spectrum of gastric disorders, including chronic atrophic gastritis (CAG), intestinal metaplasia (IM), and gastric cancer (GC) [3]. Over the past decades, extensive research has characterized multiple virulence determinants that contribute to gastric pathogenesis, including the cytotoxin-associated gene A (CagA), the vacuolating cytotoxin A (VacA), and other components encoded within the cag pathogenicity island [4,5,6,7]. Among these, CagA-positive strains are strongly associated with increased inflammation, epithelial disruption, and malignant transformation through aberrant disruption of host signaling pathways.

Although *H. pylori* infection is highly prevalent worldwide, its clinical progression exhibits pronounced heterogeneity, with severe gastric pathology or oncogenesis developing in only a subset of infected individuals [8,9]. This clinical discrepancy underscores that gastric carcinogenesis is not driven by a singular pathogenic event, but rather reflects a complex, multifactorial interplay among host genetic backgrounds, immune regulatory mechanisms, epithelial resilience, and environmental exposures. Given this multifactorial complexity, a critical priority is to identify the primary bacterial drivers that tip this delicate microenvironmental balance toward malignancy. Chief among these is the key virulence factor CagA, whose geographic divergence between East Asian and Western lineages strongly correlates with disparate global clinical outcomes [10,11]. Clinically, infection with East Asian *H. pylori* strains is associated with a significantly higher risk of severe gastric lesions, accelerated progression from CAG to IM, and ultimately a markedly elevated incidence of GC compared to Western strains. At present, contemporary clinical guidelines do not differentiate antimicrobial eradication regimens based on the lineage of *H. pylori*. However, the markedly elevated oncogenic potential of East Asian strains implies that infected individuals may benefit from strain-tailored management strategies, including intensified endoscopic surveillance and early intervention. Within the host, CagA interacts with an array of cellular targets, among which the polarity-regulating kinase MARK2 is a pivotal receptor [12,13]. Elucidating the molecular and structural divergence in how East Asian versus Western CagA binds to MARK2 is essential to deciphering their differential toxicological impacts. Crucially, it remains unmapped how these lineage-specific CagA–MARK2 interaction dynamics lead to downstream signaling aberrations, and how such molecular events ultimately reconfigure the local immune microenvironment. Resolving this cross-scale cascade requires an integrated approach that bridges high-resolution molecular dynamics with single-cell transcriptomic profiling.

Recent advances in single-cell RNA sequencing (scRNA-seq) have enabled high-resolution characterization of the immune microenvironment across various disease stages [14,15,16,17]. When applied to chronic infections, this technology provides a granular opportunity to dissect individual cell types and their associated transcriptional programs, intercellular communication networks, and immune regulatory states that drive the progressive shift from pre-malignant conditions to invasive malignancy.

In this study, we present a cross-scale, integrated framework that bridges atomistic molecular dynamics (MD) simulations with single-cell transcriptomics to dissect the gastric immune microenvironment across distinct clinical stages, including CAG, IM, and GC. By executing long-timescale MD simulations, we unveiled the structural and thermodynamic divergence between East Asian and Western CagA lineages when bound to host MARK2 kinase, identifying lineage-specific conformational dynamics that dictate binding affinity. Concurrently, utilizing a comprehensive scRNA-seq atlas of *H. pylori*–infected individuals, we mapped the global microenvironmental remodeling along the malignant cascade. Crucially, our integrated analysis revealed that a dysregulated epithelial-neural signaling axis, characterized by the progressive collapse of synaptic membranes and ion-channel transport machinery that accompanies the loss of cellular polarity, acts as a pivotal regulatory hub during disease progression. These findings provide high-resolution mechanistic insights into how molecular-level variations in biological environmental stressors propagate to drive macroscopic tissue pathogenesis. This cross-scale framework not only elucidates the toxicological basis of *H. pylori*-mediated gastric carcinogenesis, but also informs environmental health strategies for risk stratification and the prevention of pathogen-driven mucosal malignancy.

## 2. Results

### 2.1. Single-Cell Landscape of H. pylori-Associated Gastric Lesions

To comprehensively characterize the cellular heterogeneity and microenvironmental remodeling driving *H. pylori-associated* gastric carcinogenesis, we performed an integrated single-cell transcriptomic analysis across sequential pathological stages, utilizing the benchmark scRNA-seq dataset GSE249874. Following stringent quality control, cell filtering, and batch-effect correction, a total of 148,763 cells were retained for subsequent downstream analysis.

**Figure 1:**
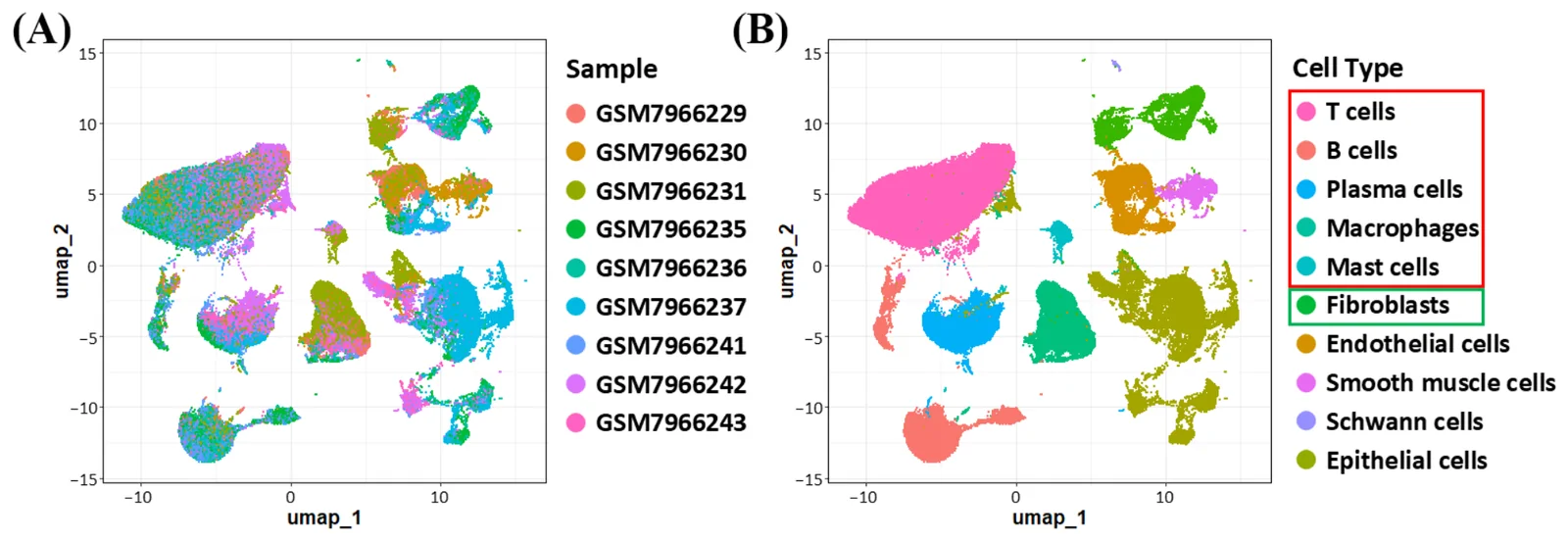
Global single-cell transcriptomic landscape of *H. pylori-infected* clinical samples. (**A**) UMAP plot of the global cellular landscape across all 9 samples, colored by individual GEO accession origin spanning three progressive disease stages, including GC samples (GSM7966229, GSM7966230, and GSM7966231), IM samples (GSM7966235, GSM7966236, and GSM7966237), and CAG samples (GSM7966241, GSM7966242, and GSM7966243). (**B**) UMAP plot of single cells profiled in the present study colored by major cell type. Lineages are grouped into functional compartments in the legend, highlighting immune compartments (red border) and stromal compartments (green border).

**Figure 2:**
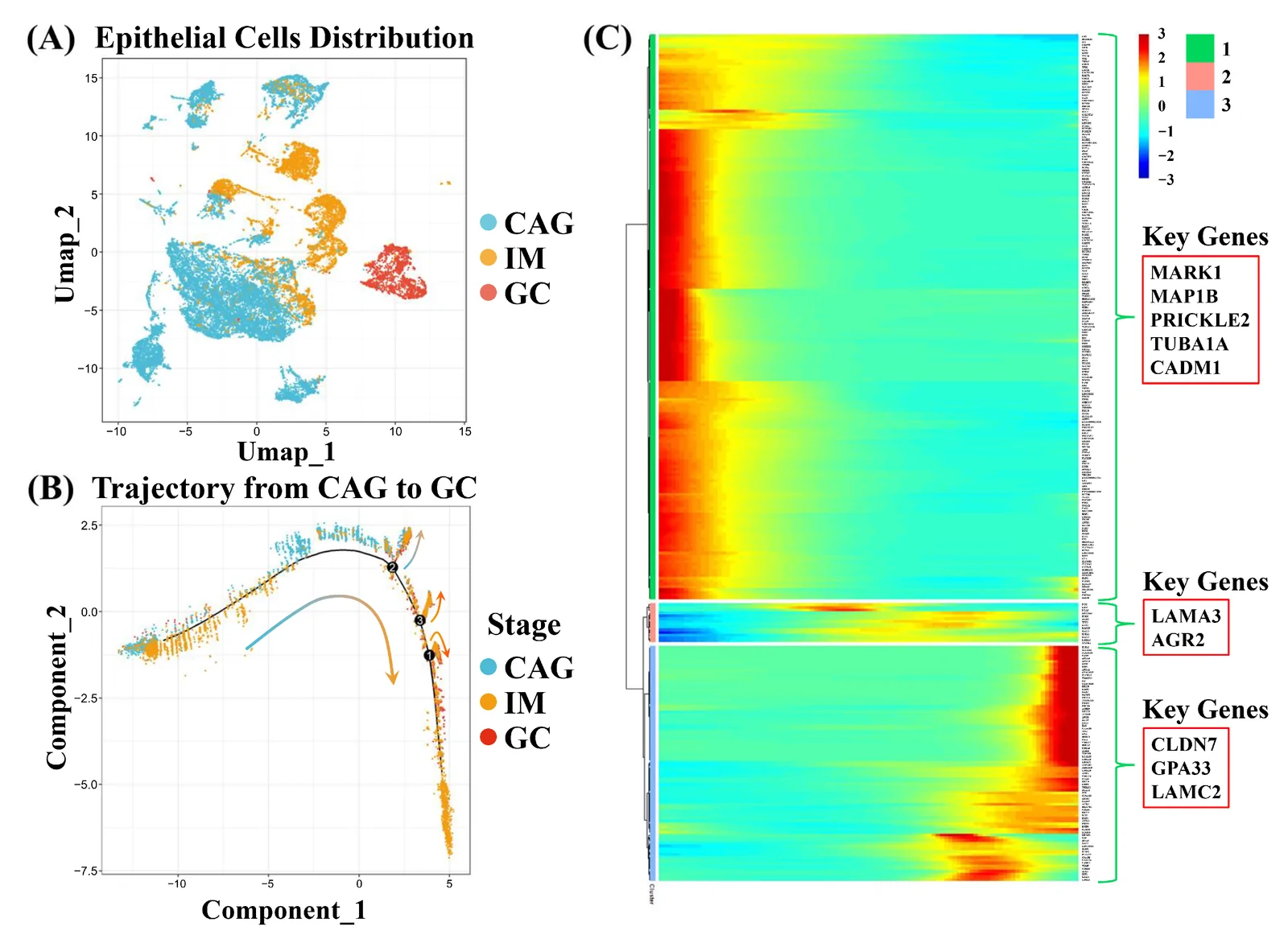
Dynamic transcriptomic characteristics of epithelial cells during gastric malignant transformation. (**A**) UMAP plot demonstrating a progressive transcriptomic shift in epithelial cells across disease stages. (**B**) Pseudotime trajectory of epithelial cells during malignant transformation. Monocle pseudotime trajectory analysis of cancer cells with highly variable genes. Cells are colored by clinical stages. The solid black line represents the multi-branched lineage backbone, with numbered circles indicating key branching nodes. (**C**) Heatmap showing the expression of representative genes across single cells along the pseudotime trajectory, grouped by gene clusters. The red boxes highlight key genes of interest within specific expression clusters.

The global distribution across all 9 samples was visualized using UMAP (Figure 1A). Unsupervised clustering and nonlinear dimension reduction via UMAP successfully segregated the global cellular landscape into ten distinct major cell types (Figure 1B). Among these cellular compartments, immune and stromal cells exhibited pronounced compositional shifts, reflecting a dynamic immune-microenvironmental reshaping in response to *H. pylori* colonization. Crucially, as epithelial cells serve as the primary cellular substrates for *H. pylori* pathogenesis and subsequent malignant transformation, this targeted compartment was further isolated and stratified based on disease progression stages. This targeted analysis uncovered a distinct spatial segregation along the pathological axis of CAG, IM, and GC (Figure 2A).

### 2.2. Lineage Divergence and Temporal Gene Expression Clustering of Gastric Epithelial Cells

To delineate the dynamic transcriptional transitions and progressive cell-fate trajectories of gastric epithelial cells during *H. pylori*–driven malignant transformation, we conducted semi-supervised pseudotime trajectory analysis utilizing Monocle2 (Figure 2B). The inferred evolutionary roadmap originated within the CAG state and advanced through a series of sequential branching nodes, revealing a complex, multi-branched lineage topology rather than a simple linear continuum.

This nonlinear structure highlights the highly heterogeneous evolutionary paths of the compromised epithelium. Following the early lineage divergence, the trajectory enters a dense cluster of closely spaced branching events centered around nodes 2, 3, and 1. Rather than representing separate chronological stages, these proximate nodes define a volatile evolutionary hot zone of transitional epithelial cells. At this intersection, these minimally differentiated and transcriptomically similar cells stand at the precipice of malignant transformation under persistent stress driven by pathogens. While one sub-lineage progresses toward a committed, terminal IM state at the elongated tail, parallel intermediate cells in the exact same temporal window abruptly abort this normal differentiation path, segregating instead toward malignancy. This concentrated branching at nodes 1–3 underscores the profound lineage plasticity of transforming epithelial cells. Rather than a gradual, linear progression, the dense packing of these bifurcation nodes marks a critical transitional threshold. Within this identical pseudotime window, the co-existence of both IM and GC cells demonstrates that a single population of unstable, transitional epithelium can rapidly bifurcate into dual, divergent pathological outcomes, whereby cells either track the terminal differentiation path toward specialized IM or segregate directly toward GC.

To decode the temporal molecular programs governing these lineage-fate transitions, we identified pseudotime-dependent differentially expressed genes (DEGs) along the evolutionary axis. By employing unsupervised kinetic clustering, these chronologically altered transcripts were stratified into three major co-expression modules based on their distinct expression wave patterns across the pseudotime continuum (Figure 2C and Table S1). Among these, Cluster 1 displayed a remarkable, sharp downregulation exclusively at the late phase of the trajectory, whereas Cluster 3 exhibited an upregulation. This specific dynamic pattern highlights that genes within Cluster 1 are progressively silenced or severely compromised during terminal epithelial transformation and malignant progression, signifying a profound erosion of homeostatic epithelial identity and baseline mucosal functions that precedes late-stage gastric carcinogenesis.

### 2.3. Epithelial Polarity Collapse Triggers Neuroendocrine-like Secretory and Synaptic Programs in Late-Stage Transformation

**Figure 3:**
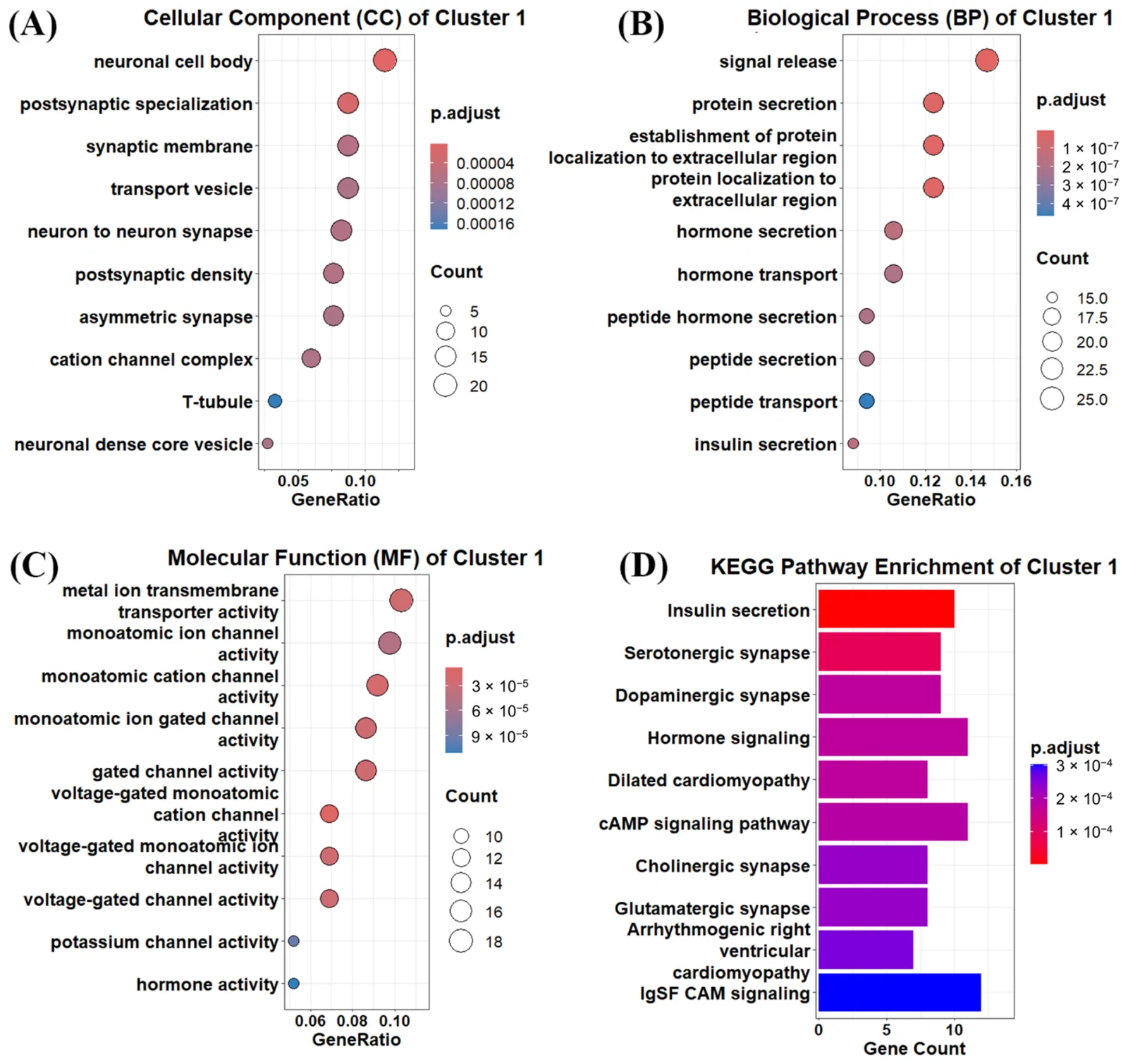
Gene Ontology and KEGG pathway enrichment profiles of Cluster 1 genes. (**A**) Cellular Component terms. (**B**) Biological Process terms. (**C**) Molecular Function terms. (**D**) Bar plot illustrating the KEGG pathways.

To systematically uncover the functional landscape governing downstream epithelial transformation, we executed comprehensive GO and KEGG functional enrichment analyses targeting the temporally dynamic gene signatures. Although the upregulated genes in Cluster 3 naturally aligned with gastric cancer progression (Figure S1), an unexpectedly rich and complex functional landscape emerged from Cluster 1. On a structural and physiological level, the progressive downregulation of these trajectory-defining transcripts revealed a severe dissolution of normal cellular architecture, with the compromised epithelium showing a pronounced depletion of structural components characteristic of highly specialized, neuroendocrine-like epithelial networks, including the neuronal cell body, postsynaptic specialization, and synaptic membrane (Figure 3A). Crucially, this structural and electrochemical dismantling directly led to the impairment of directional exocytosis, as evidenced by the high-intensity enrichment of baseline terms associated with signal release and hormone or peptide secretion pathways (Figure 3B). Functionally linked to these deteriorating domains was a concurrent enrichment of foundational electrochemically active machineries, notably gated channel activity and canonical neurotransmission cascades spanning serotonergic, dopaminergic, glutamatergic, and cholinergic synapses (Figure 3C,D). Together, this interconnected transcriptomic signature provides robust evidence that during nonlinear transformation, CagA-mediated epithelial polarity collapse fundamentally subverts pre-existing epithelial networks by shutting down homeostatic synaptogenesis and vesicle fusion cascades, marking a catastrophic loss of functional baseline defenses during gastric carcinogenesis.

### 2.4. Progressive Remodeling of the Gastric Tumor Microenvironment

**Figure 4:**
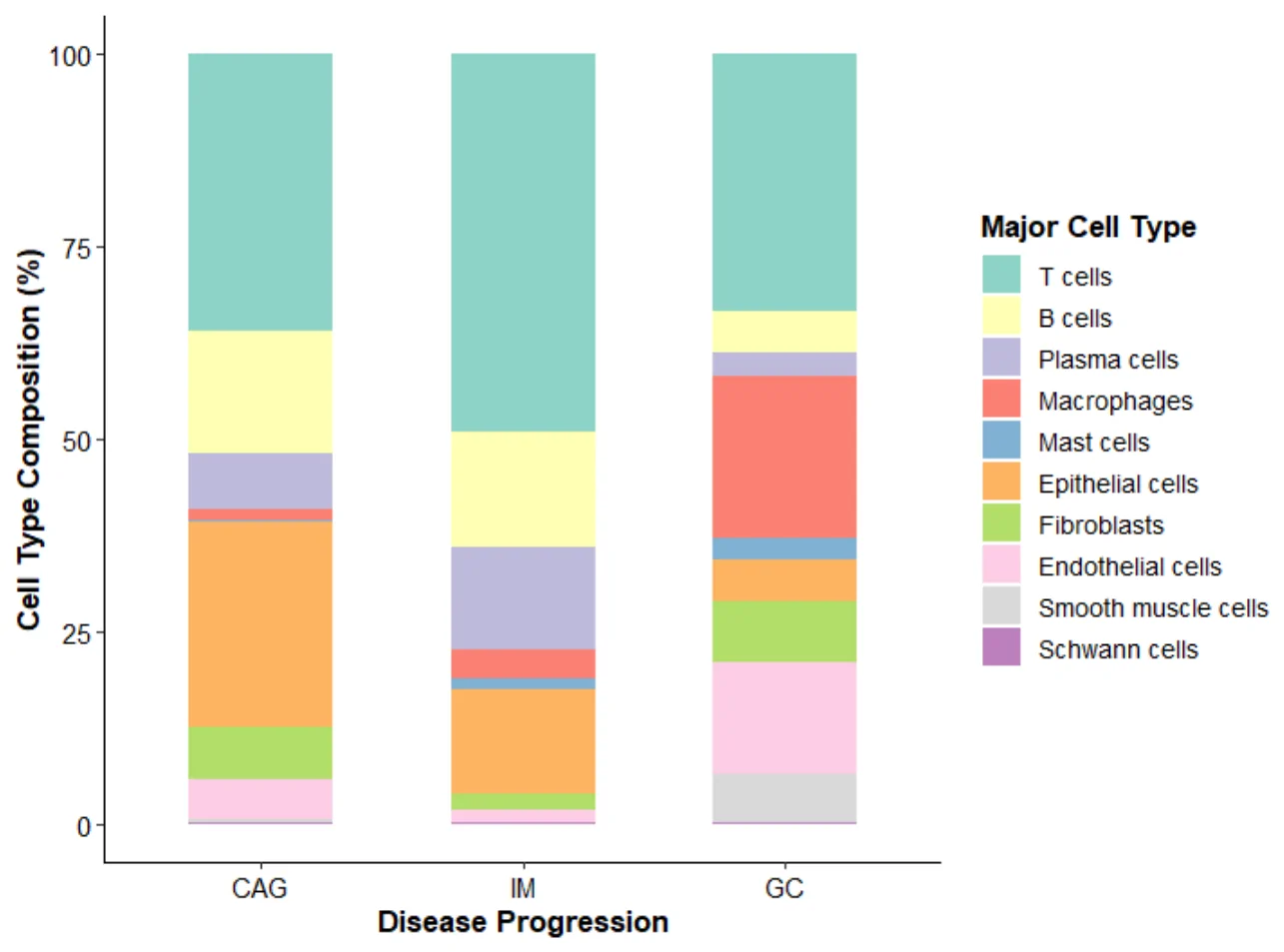
Dynamic remodeling of cellular composition ratios across gastric pathological stages. Relative proportions of annotated single-cell lineages across progressive disease stages, including CAG, IM, and GC. Each stacked column represents 100% of the single-cell transcriptomic profile for the indicated stage, color-coded by major cell lineages as specified in the legend.

To delineate the microenvironmental evolution during gastric carcinogenesis, we quantified cell-type composition ratios across the CAG, IM, and GC continuum (Figure 4). Proportional profiling revealed that the lesion ecosystem undergoes a profound cellular shift, characterized primarily by the progressive enrichment of fibroblasts and a dramatic, explosive expansion of macrophages exclusively within the GC stage. Conversely, T cell populations exhibited dynamic compositional fluctuations that peaked during the intermediate phases. Specifically, T cell proportions expanded markedly during the IM stage, peaking before declining in the GC stage. This dynamic transient expansion in IM likely reflects a heightened adaptive immune response and localized immune surveillance triggered by persistent *H. pylori* antigenic stimulation and active mucosal intestinalization. However, as lesions transition into GC, the microenvironment undergoes immunosuppressive rewiring, characterized by the explosive recruitment of macrophages and stromal components, which diminishes the relative proportion of T cells and suppresses their local immune functional efficacy, thereby facilitating potential immune evasion. Rather than representing isolated shifts, these altered cell-type proportions fundamentally reshape the spatial proximity and structural density of the transforming niche.

**Figure 5:**
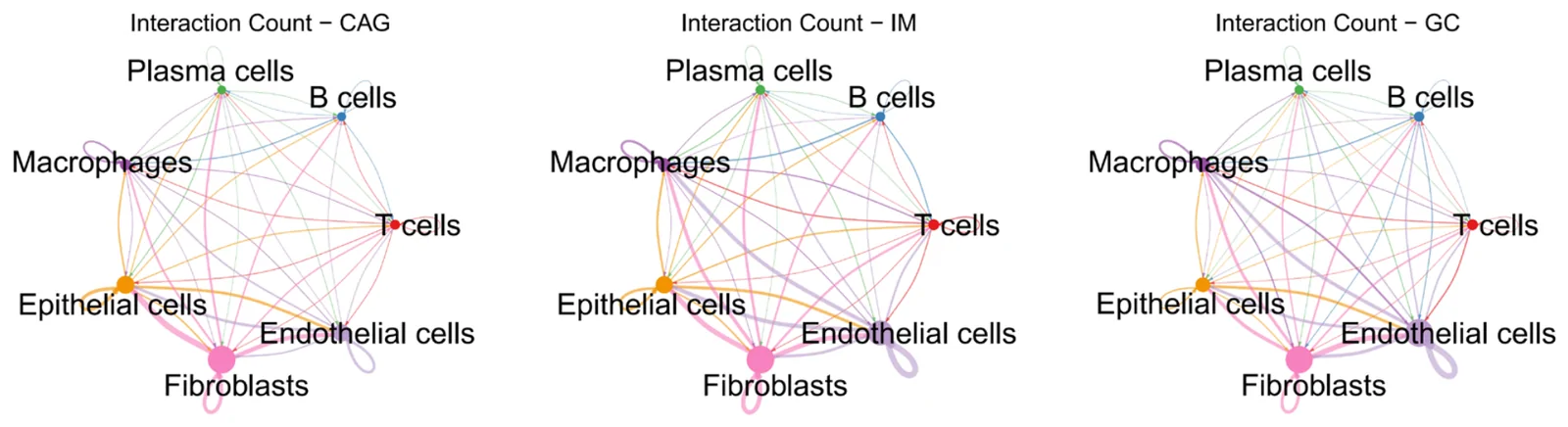
Cellular communication networks during disease progression. Global interaction networks depicting cellular communication counts across progressive disease stages, including CAG, IM, and GC. Circle nodes represent distinct cell lineages, with node size proportional to relative cell abundance. Colored directed edges denote ligand–receptor signaling interactions between source and target cell populations, where line thickness corresponds to interaction count and signal strength.

Concurrently, to resolve how these compositional macro-shifts dynamically rewire the underlying intercellular signaling circuitries, we performed comparative cell–cell communication analysis utilizing CellChat to map the global interaction networks across the disease spectrum (Figure 5). The computed total interaction strength revealed highly specialized, stage-specific rewiring of cellular dialogues. Intriguingly, the crosstalk between fibroblasts and epithelial cells peaked during the pre-malignant CAG and IM phases, where normal fibroblasts extended dense, intensive communication threads to epithelial cells, a process that molecularly aligns with the hyperproliferative, active regenerative repair cascades of damaged mucosa prior to malignant transformation. The underlying ligand–receptor pairs driving these cellular dialogues for both fibroblasts and T cells are detailed in Figures S2 and S3, respectively.

### 2.5. Structural Modeling of Western and East Asian CagA

**Figure 6:**
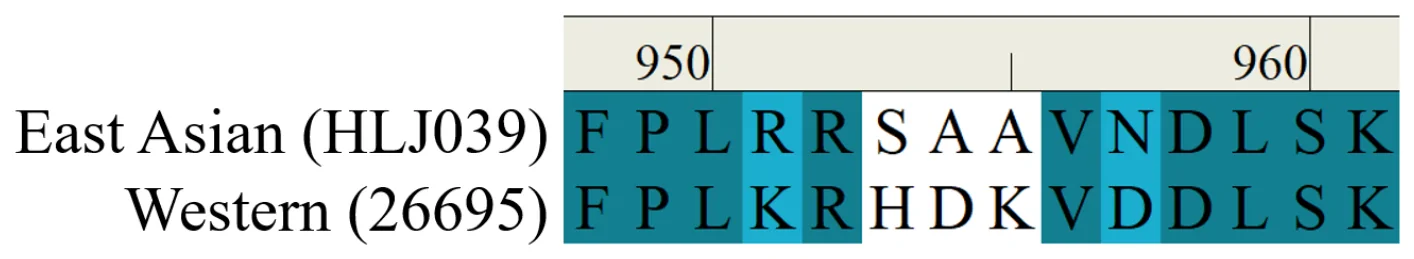
Sequence alignment of the 14 amino acid MARK2 interacting region within the CagA from East Asian (*H. pylori* strain HLJ039) and Western (*H. pylori* strain 26695) lineages. Shading intensity indicates the level of sequence similarity, with darker blue representing higher conservation.

Given that our transcriptomic profiling unmasked a catastrophic collapse of polarized epithelial networks presumably driven by CagA, we sought to decipher the precise structural blueprint underlying the CagA–MARK2 interaction across geographically distinct strains. Consequently, we performed comparative sequence alignment within their conserved MARK2-binding domains (Figure 6). This computational alignment revealed a highly localized yet physicochemically distinct substitution hotspot embedded within the core recognition motif.

Specifically, the Western CagA lineage possesses an H-D-K (Histidine-Aspartate-Lysine) sequence characterized by large, highly polar, and electrically charged side chains. In contrast, the East Asian lineage undergoes a drastic transition to a neutral, small-sized S-A-A (Serine-Alanine-Alanine) motif. This marked loss of charged residues and alteration in local stereochemistry are highly likely to remodel the surface electrostatic potentials and modulate the binding affinity and spatial stability with host MARK2.

### 2.6. Molecular Dynamics Simulation Reveals Lineage-Specific Binding Stability

**Figure 7:**
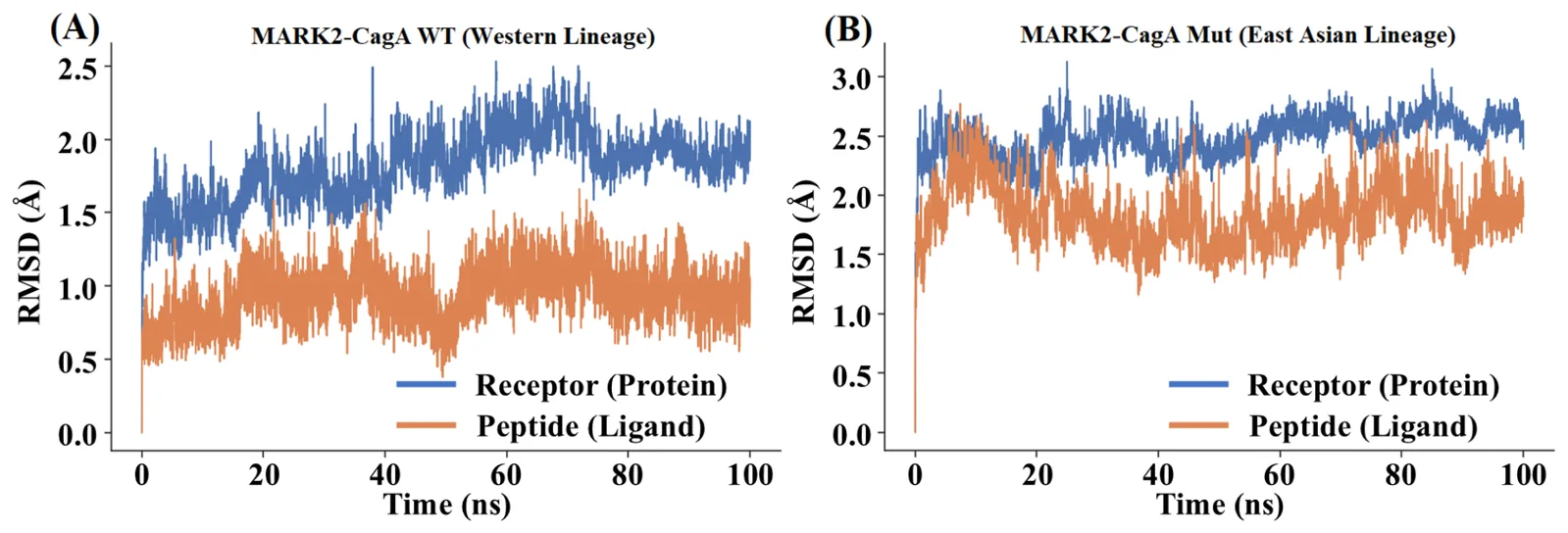
RMSD trajectories of MARK2–CagA complexes during 100 ns MD simulations. (**A**) The Western system designated as WT (MARK2-CagA WT). (**B**) The East Asian system designated as Mut (MARK2-CagA Mut).

To evaluate the structural stability and dynamic behavior of the MARK2 protein in complex with different CagA isoforms, 100 ns MD simulations were performed (Figure 7). The Root Mean Square Deviation (RMSD) was calculated for both the receptor (MARK2) and the peptide ligand (CagA) to assess the convergence and conformational equilibrium of the systems, with the Western lineage complex designated as WT (Wild Type) and the East Asian lineage complex as Mut (Mutant) for comparative trajectory tracking.

In the Western CagA–MARK2 system, the receptor exhibited a steady increase in RMSD during the first 20 ns, eventually reaching a plateau around 1.5–2.0 Å. The peptide ligand demonstrated significant rigidity, with RMSD values consistently remaining below 1.5 Å throughout the majority of the 100 ns trajectory. These relatively low and steady fluctuations suggest that the Western lineage complex maintains a highly rigid and stable binding pose after initial equilibration.

In contrast, the East Asian lineage complex showed increased conformational flexibility. While its MARK2 receptor reached a plateau at a similar timescale, it exhibited slightly higher average RMSD values, fluctuating between 2.0–2.7 Å. Notably, the peptide ligand within this East Asian system displayed more pronounced fluctuations compared to its Western counterpart, with RMSD values often exceeding 2.0 Å and showing a broader range of motion.

A comparison of the two systems indicates that while both complexes reached a state of relative equilibrium within 100 ns, the East Asian lineage introduces greater dynamic instability into the binding interface. The increased RMSD of the peptide (quantified based on the positional distance deviations of backbone heavy atoms relative to the initial structure) in the Mut system suggests a more flexible binding mode, which may be associated with the enhanced pathogenic potential or altered signaling interference characteristic of East Asian CagA strains.

**Figure 8:**
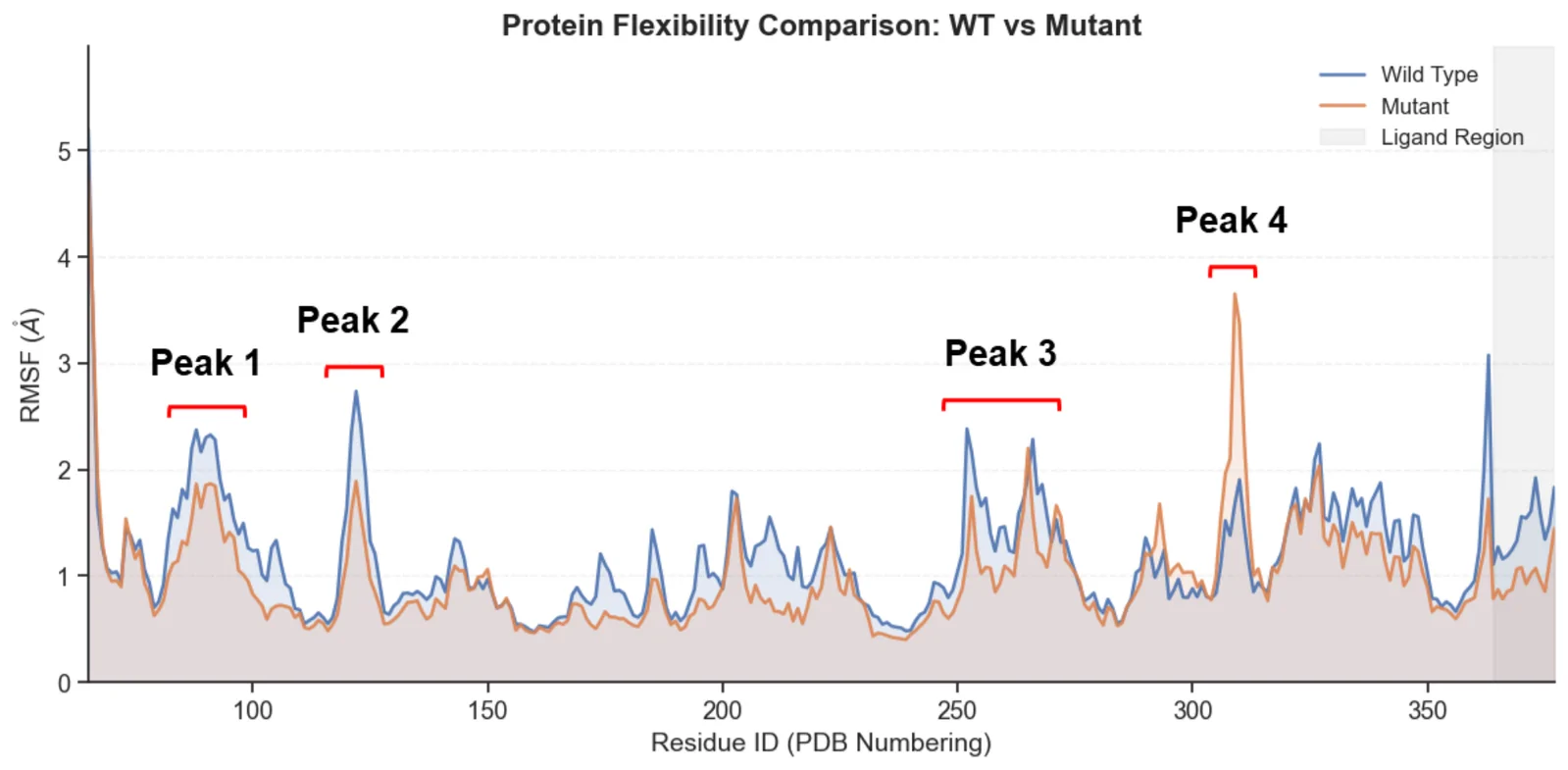
RMSF profiles illustrating the comparative residual flexibility of MARK2 amino acid residues in complex with Western lineage and East Asian lineage CagA isoforms. Prominent flexibility peaks are annotated to highlight regional dynamic disparities: Peaks 1–3 (residues 90–100, 120–130, and 250–270, respectively) denote distinct regions where the Western lineage complex (WT) exhibits elevated basal flexibility compared to its East Asian counterpart; Peak 4 (residue 310) highlights a sharp, mutant-specific flexibility spike in the East Asian complex (Mutant). The grey shaded region on the right delineates the peptide ligand segment.

To pinpoint the regions contributing to the differences in conformational dynamics between the two complexes, Root Mean Square Fluctuation (RMSF) was calculated for each residue of the MARK2 receptor across the 100 ns trajectory (Figure 8). In general, both the WT and Mut systems exhibited low fluctuations (<2.0 Å) within the structured core regions of the protein, indicating a preserved overall fold. However, distinct, lineage-specific patterns of flexibility were observed.

Compared to the East Asian lineage (Mut) complex, the Western lineage (WT) MARK2 complex displayed higher overall basal flexibility, with several notable peaks. Specific regions, particularly around residues 90–100, 120–130, and a region just after residue 250, showed elevated RMSF values, indicating a greater range of movement in these loops or domains in the presence of the Western lineage peptide. Conversely, the Mut complex generally exhibited attenuated fluctuations across the majority of the sequence, suggesting that its interaction exerts a global rigidifying effect on the MARK2 dynamic scaffold. The sole exception was a sharp, prominent flexibility spike around residue 310, where the Mutant RMSF approached 4.0 Å, significantly exceeding the corresponding WT counterpart.

Interestingly, despite these localized deviations, the fluctuation profiles within the defined Ligand Binding Region remained remarkably congruent between both lineages. This stark contrast suggests that the lineage-specific functional differences and affinity shifts are likely mediated by alterations in distal dynamic coupling or allosteric wiring, rather than drastic, localized conformational rewiring directly at the primary binding interface.

### 2.7. Thermodynamic Basis of CagA-MARK2 Interaction via MM/GBSA

To quantitatively compare the binding affinities of the Western and East Asian CagA lineages toward the host MARK2 receptor, the total binding free energy (Δ*G_total_*) and its individual thermodynamic components were determined using the MM/GBSA method (Figure S4). Trajectory-wide energetic profiling indicated that the East Asian (Mut) complex exhibited a more favorable, lower binding free energy (Δ*G_total_* = −85.68 ± 6.30 kcal/mol) compared to the Western (WT) counterpart (Δ*G_total_* = −80.99 ± 5.02 kcal/mol). This thermodynamic disparity indicates that the East Asian CagA isoform possesses a higher binding affinity for MARK2, potentially underpinning its enhanced pathogenic activity observed clinically. A detailed analysis of the energy components revealed that electrostatic interactions (Δ*G_elec_*) and van der Waals forces (Δ*G_vdw_*) are the primary driving forces for complex formation in both systems. Interestingly, while the Western lineage (WT) showed a significantly stronger electrostatic attraction (Δ*G_elec_* = −584.32 kcal/mol) than the Mutant variant (Δ*G_elec_* = −360.69 kcal/mol), this favorable contribution was largely counteracted by a much higher polar solvation energy penalty (Δ*G_GB_* = 623.16 kcal/mol for WT compared with 391.27 kcal/mol for Mut). This elevated desolvation penalty in the WT system arises because highly polar or charged interfacial residues must strip off their surrounding hydration shells upon binding, incurring a substantial thermodynamic cost. Notably, the reduction in polar solvation penalty in the East Asian variant (ΔΔ*G_GB_* = −231.89 kcal/mol) fully offsets its attenuated electrostatic attraction (ΔΔ*G_elec_* = +223.63 kcal/mol), yielding a more favorable net polar contribution (Δ*G_elec_* + Δ*G_GB_* = +30.58 kcal/mol for Mut compared with +38.84 kcal/mol for WT). In contrast, the East Asian CagA isoform achieved a more optimal thermodynamic balance. Although the absolute electrostatic contribution was attenuated, a substantially reduced desolvation cost ultimately allowed this variant to achieve a stronger net binding affinity. The nonpolar solvation energy (Δ*G_SA_*) and van der Waals contributions remained relatively comparable between the two lineages, though slightly more pronounced in the WT system. These biophysical insights demonstrate that the lineage-specific sequence divergence modulates MARK2 binding affinity primarily by optimizing the electrostatic–solvation energy trade off at the intermolecular interface.

### 2.8. Binding Energy Decomposition Reveals Heterogeneous Interaction Motifs

**Figure 9:**
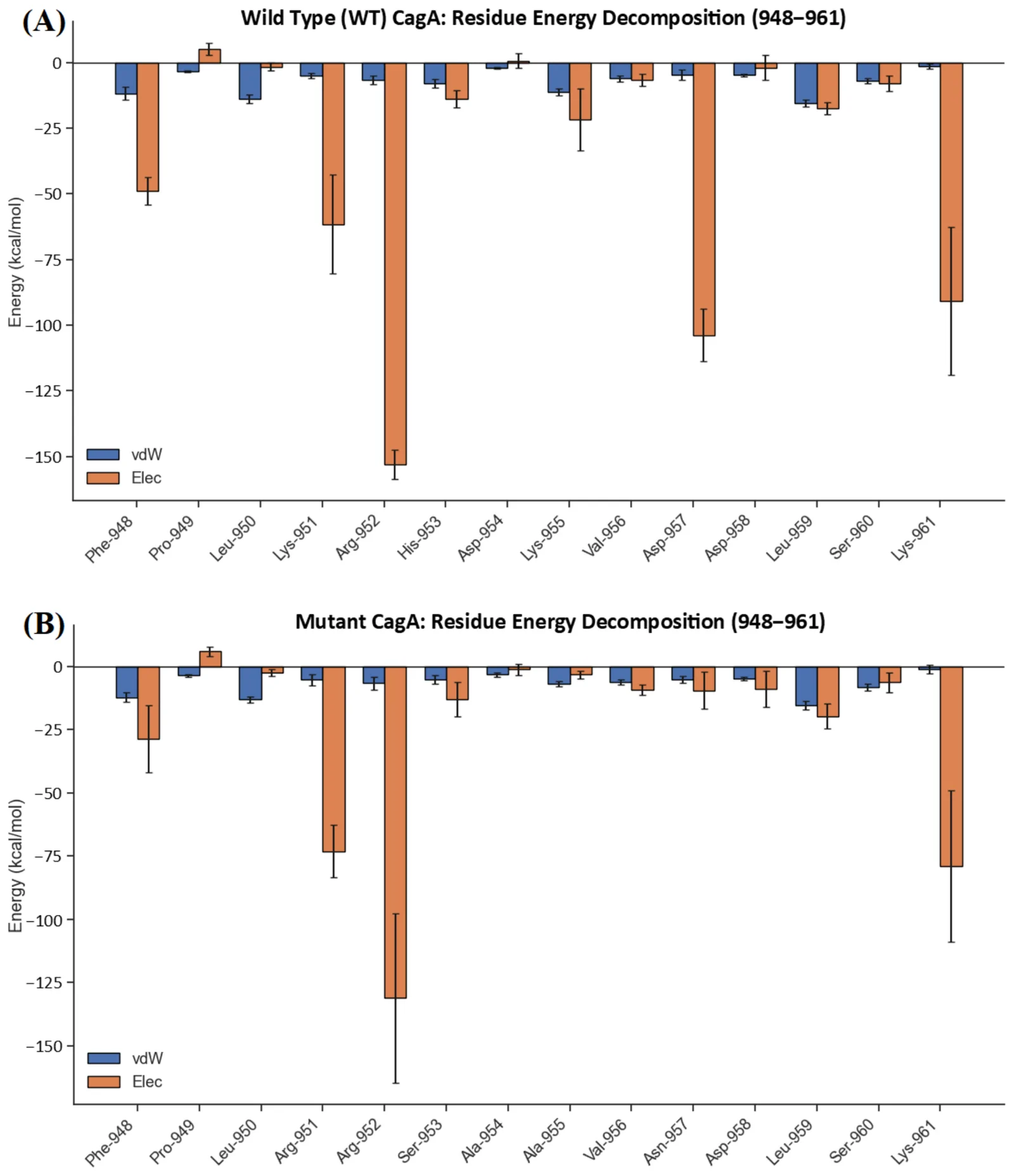
MM/GBSA per-residue binding energy decomposition of Western and East Asian CagA peptide segments. (**A**) Energetic decomposition of the Western lineage CagA. (**B**) Energetic decomposition of the East Asian lineage CagA.

To quantify the energetic contribution of individual residues to the overall binding free energy, MM/GBSA per-residue energy decomposition was performed for the CagA peptide segments (residues 948–961) in both complexes. The analysis revealed that electrostatic interactions serve as the primary driving force for CagA–MARK2 docking, featuring a highly heterogeneous distribution of energetic contributions across the peptide primary sequences (Figure 9).

As the most prominent thermodynamic feature, Arg952 contributed the highest absolute electrostatic energy to the complex in both lineages, reaching approximately −150 kcal/mol in the WT system and −125 kcal/mol in the Mut system. This dominant energy contribution identifies Arg952 as the indispensable electrostatic anchor residue for complexation across both strains.

A direct comparison between the two lineages highlighted distinct energetic fingerprints. The Western CagA peptide utilizes a broader, more distributed network of strongly contributing residues, including Lys951, Asp957, and Lys961, all of which provided substantial electrostatic stabilization, with Asp957 notably exceeding −100 kcal/mol. This suggests a well-distributed and cooperative binding interface.

In contrast, the East Asian CagA peptide showcased a more centralized and concentrated energy profile. However, this localized thermodynamic gain was offset by a dramatic attenuation in the energy contribution of Asp957, which became substantially diminished in the East Asian complex. This highly localized intensification of binding energy, combined with the loss of distal electrostatic anchors like Asp957, may result in a more topologically constrained yet dynamic binding mode. This unbalanced energetic weighting perfectly corroborates the larger RMSD fluctuations observed previously for the East Asian lineage during the dynamic trajectories.

### 2.9. Structural Basis of the MARK2-CagA Interface Post-MD Simulation

**Figure 10:**
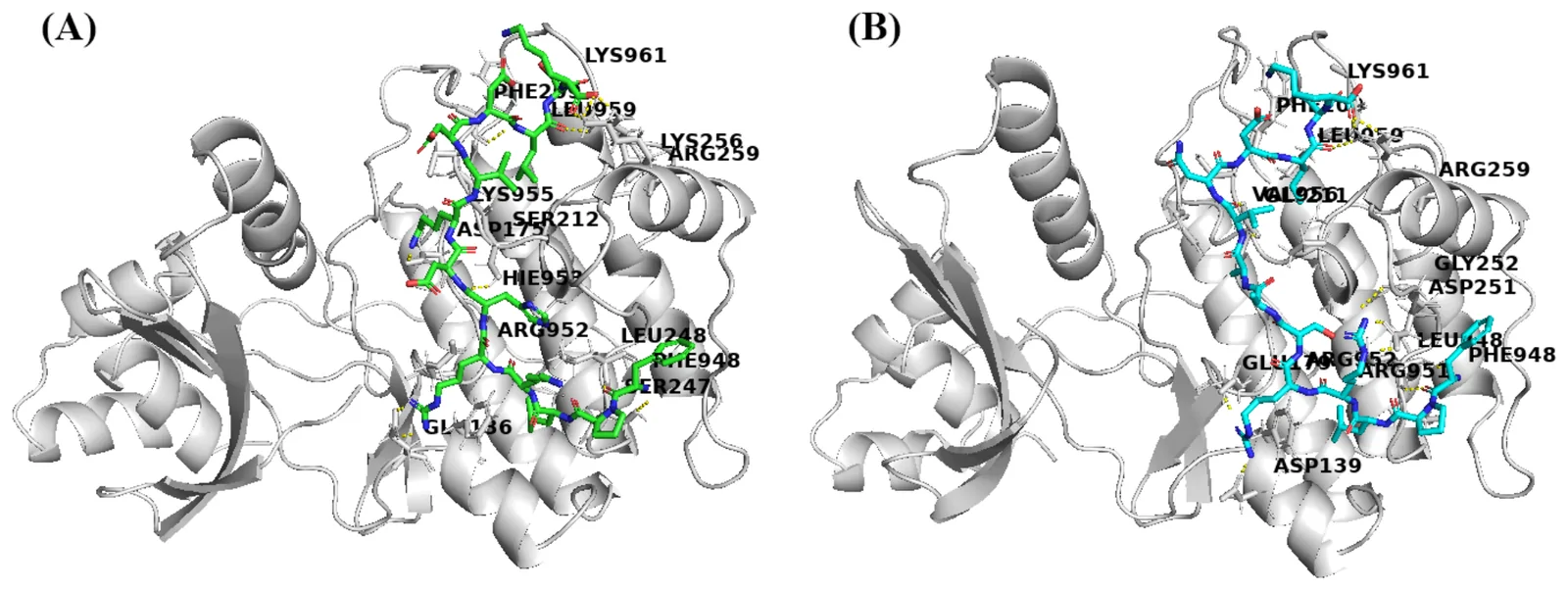
Structural interactions within MARK2–CagA complexes. (**A**) Interface of the MARK2–Western CagA complex. (**B**) Interface of the MARK2–East Asian CagA complex.

Equilibrium trajectory analysis confirmed that both CagA lineages target a conserved MARK2 pocket, driven by the indispensable anchor residue Arg952, which forms persistent interaction with MARK2 acidic cluster. However, a defining physicochemical reconfiguration occurs at the flanking residues. While the Western lineage relies on a distributed (Figure S5), solvent-exposed polar network (e.g., Phe196-Lys955, 33.9%), the Mut complex features a strategic enrichment of localized hydrophobic interactions, most notably the Leu248-Phe948 pair (22.9%). As visually captured by the post-MD PDB structures, these bulkier hydrophobic side chains within the SASA induce a localized “hydrophobic expulsion” that excludes water molecules. This drastically minimizes the polar solvation penalty (Δ*G_GB_*), mechanistically explaining how the East Asian lineage achieves higher net affinity with a lower desolvation cost, despite its higher peripheral dynamic fluctuations. This structural reconfiguration and specific residue-level interactions for both complexes are comprehensively shown in Figure 10.

## 3. Discussion

By leveraging single-cell transcriptomics across progressive clinical stages, this study delineates the cellular topography of *H. pylori-associated* gastric carcinogenesis in East Asian populations. Our analysis highlights that epithelial transformation is intimately coupled with systemic microenvironmental flux. During early gastritis and metaplasia stages, fibroblasts exhibit highly pronounced interaction strengths with epithelial cells, acting as critical stroma-priming agents that destabilize the epithelial niche. This cellular priming, paired with a late-stage remodeling of the T-cell-mediated immune microenvironment, establishes an immunosuppressive, pro-tumorigenic sanctuary that fosters unconstrained epithelial reprogramming. Regarding the microenvironmental dynamics during disease progression, our intercellular communication analysis captured a transient expansion of T cell-epithelial signaling during the IM stage followed by a notable contraction in GC. This transient enhancement aligns with the documented induction of localized immune surveillance during pre-malignant epithelial remodeling, where T cell-mediated responses are actively recruited to counter early dysplastic alterations [18,19]. However, upon progression to the GC stage, this T cell-epithelial communication network notably contracted. This reduction in signaling density suggests a potential onset of immune evasion, a hallmark event where established malignant cells disrupt homeostatic intercellular dialogues and suppress effective T cell engagement to bypass microenvironmental clearance [20,21].

Located within this evolving niche, the gastric epithelium undergoes profound phenotypic rewiring driven by lineage-specific pathogen stress. Pseudotime trajectory and pathway enrichment clustering reveal that the East Asian CagA–induced collapse of MARK2-mediated architectural polarity directly triggers an aberrant transdifferentiation toward neuroendocrine-like secretory and synaptic communication programs [22,23]. From a mechanistic perspective, this anomalous expression of polarity-dependent synaptic and secretory features strongly implies a molecular subversion of homeostatic epithelial networks. In healthy epithelium, directional vesicle trafficking and membrane domain specialization are strictly governed by a core network of polarity-regulating factors, including the kinase MARK2, which functions as a key gatekeeper of microtubule stability and asymmetric protein distribution [24,25]. Given that the *H. pylori* virulence factor CagA directly binds and inhibits host MARK2 to dismantle cell polarity, our transcriptomic findings provide a compelling functional linkage. Pathological disruption of this regulatory machinery by CagA likely triggers severe microtubule destabilization and cell polarity rewiring, forcing the anomalous cargo sorting that mimics neurosecretory exocytosis [26,27]. Biologically, this is not an evolutionary coincidence. The molecular machinery governing epithelial polarized vesicle trafficking shares a deep ontological blueprint with the synaptic vesicle cycle. The physical demise of cell polarity reconfigures the intracellular trafficking networks, aberrantly hijacking synaptic-like vesicle fusion and neuroactive ligand–receptor cascades. Transcriptionally, this programmatic plasticity equips pre-malignant clones with heightened survival and autonomous signaling capacity, successfully bridging stroma primed microenvironmental cues with catastrophic epithelial cell fate transitions [28,29].

To decipher the molecular mechanism responsible for this host epithelial polarity disintegration, we examined the energetic components governing the CagA–MARK2 complexation. Although clinical consensus associates East Asian *H. pylori* strains with an elevated risk of gastric malignancy, the underlying biophysical principles have remained less clear. Our thermodynamic component analysis resolves this by revealing a pivotal energetic trade off, specifically, a delicate balance between gas phase electrostatic attraction and polar desolvation penalty, that dictates lineage-specific binding performance.

Global free energy evaluation demonstrates that the East Asian CagA peptide possesses a more favorable net binding affinity toward host MARK2 than the Western lineage [30,31]. Crucially, this superior affinity does not stem from an enhancement of absolute electrostatic attractions. Rather, the Western lineage exhibits a much stronger gas phase electrostatic attraction, which is, however, thoroughly offset by a prohibitive polar solvation penalty. This indicates that the Western CagA–MARK2 interface is over-engineered for polar interactions, sacrificing net affinity due to the extreme energetic cost required to displace water molecules from the pocket. Conversely, the East Asian lineage achieves a superior thermodynamic optimum. By accepting a moderate reduction in absolute electrostatic pull, it drastically minimizes the local desolvation penalty. This optimized affinity solvation compromise allows the East Asian lineage to achieve a more favorable net binding free energy, providing a firm biophysical rationale for its enhanced thermodynamic tenacity.

By scaling down from global thermodynamics to residue-level interactions, our per-residue energy decomposition and contact occupancy analysis resolve the apparent paradox of how the East Asian peptide can maintain a higher overall binding affinity while concurrently exhibiting larger trajectory-wide RMSD fluctuations. The Western CagA peptide utilizes a distributed, cooperative network of polar and electrostatic anchors (including Lys951, Asp957, and Lys961) that rigidly pins the peptide across the entire interface, minimizing RMSD fluctuations but incurring massive desolvation costs.

In contrast, the East Asian lineage adopts a more centralized, localized interlocking mode that relies on strategic hydrophobic clustering. At the core of this configuration, the synergy between the common anchor Arg952 and the lineage-specific hydrophobic pairing of Phe948 shifts the structural balance from rigid flatness to a highly resilient, dynamically tethered mode. Post-MD conformational evaluation suggests that the bulkier hydrophobic side chains within the SASA act as a spatial shield, creating a localized hydrophobic expulsion that insulates the binding pocket. This structural shield localized the desolvation penalty to a minimized pocket area. Consequently, while the absence of distal polar anchors permits the peripheral segments of the East Asian CagA peptide to remain highly flexible, thereby explaining the elevated distal RMSD, the core pathogenic motifs maintain a highly persistent contact occupancy that locks the primary MARK2 catalytic site.

This structural locking provides a direct, causal bridge to the cellular disruptions identified in our scRNA-seq analyses. By competitively and persistently binding to the MARK2 kinase domain, the East Asian CagA peptide profoundly disrupts its ability to phosphorylate downstream targets, triggering the catastrophic disassembly of tight junctions and the subsequent collapse of epithelial polarity. This persistent molecular blockade accelerates the phenotypic rewiring toward neuroendocrine-like synaptic programs, directly explaining why populations exposed to East Asian *H. pylori* strains experience significantly accelerated mucosal transformation and elevated GC risks [32,33]. At the atomistic scale, this lineage-specific structural interlocking provides a compelling molecular link to our transcriptomic pathway enrichment analysis. By capturing the host MARK2 kinase domain with higher thermodynamic tenacity and prolonged contact occupancy, the East Asian CagA peptide exerts a more sustained and potent functional perturbation on this master polarity regulator. This persistent molecular blockade severely deranges downstream polarity signaling, driving tight junction disassembly and epithelial rewiring. These micro-level structural dynamics mechanistically rationalize why patients infected with East Asian *H. pylori* strains present with a significantly heightened risk of GC, successfully bridging computational biophysics with the epidemiological reality.

Beyond biophysical mechanics, these lineage-specific binding differences hold profound clinical implications. Traditional *H. pylori* eradication relies on broad-spectrum antibiotics, which frequently drive treatment resistance and disrupt gut microbiota balance. By pinpointing the unique structural interface between East Asian CagA and host MARK2, our study provides a concrete structural blueprint for precision anti-virulence therapeutics. Specifically, the lineage-specific hydrophobic pocket anchored by Phe948 presents a rationale for structure-based drug design. Peptidomimetic decoys or small-molecule protein–protein interaction inhibitors can be engineered to specifically fit this interface, competitively blocking the CagA–MARK2 docking site or allosterically neutralizing the inhibitory effect of CagA on the MARK2 kinase domain. By rescuing host MARK2 function without exerting lethal selection pressure on the bacterium, such strain-tailored anti-virulence agents would selectively ablate the primary oncogenic driver while preserving host gut microbiota. Furthermore, these sequence-dependent mechanical insights support strain-level patient stratification, allowing clinicians to identify high-risk individuals infected with high-affinity CagA variants for early, targeted endoscopic surveillance and personalized intervention.

By integrating single-cell transcriptomics with biophysical MD simulations, this study establishes an integrative computational framework that tracks the cytotoxic mechanisms of *H. pylori* CagA from macroscopic cellular landscapes down to atomic coordinates. This combined strategy bridges the traditional gap between computational genomics and structural biology, providing a comprehensive, complementary view of CagA-induced host virulence and tissue niche remodeling.

Despite these insights, several limitations must be acknowledged. First, the structural modeling and dynamic trajectories were executed utilizing a 14 amino acids peptide encompassing the core functional MARK2-binding motif of CagA. While this short peptide encapsulates the core functional motif interacting with MARK2, future long-scale simulations using the full-length 3D structure will be required to capture potential long-range domain movements. Second, although our transcriptomic and structural findings are robust and internally consistent, they remain predictive in nature. Extensive in vitro kinase activity assays and functional in vivo validations are warranted in future studies to experimentally consolidate the precise residue-level interaction networks and the downstream neuroendocrine-like differentiation dynamics identified herein.

## 4. Materials and Methods

### 4.1. Single-Cell RNA Sequencing Data Collection

Publicly available scRNA-seq data of human gastric lesion tissues were retrieved from the NCBI Gene Expression Omnibus (GEO) repository under accession number GSE249874 [34]. To control for biological confounding factors, strict inclusion criteria were applied requiring that all selected specimens possess clinically confirmed *H. pylori* infection. Consequently, a total of 9 Chinese patients representing three distinct pathological stages along the gastric carcinogenesis cascade were included in this study, evenly stratified across three progressive pathological stages including CAG (*n* = 3), IM (*n* = 3), and GC (*n* = 3). To ensure standardized data quality, raw sequencing reads in SRA format (SRR files) were downloaded from the Sequence Read Archive. The raw fastq files were then processed using the Cell Ranger pipeline (v10.0.0, 10x Genomics) for alignment against the human reference genome (GRCh38), cell barcode identification, and UMI counting.

### 4.2. Single-Cell Data Preprocessing and Quality Control

Downstream bioinformatic analysis was performed using the Seurat package (version 4.3.0), where individual patient objects were initially merged into a unified dataset. To exclude low-quality or dying cells, barcodes were filtered to retain only those containing 500 to 6000 detected genes and less than 15% mitochondrial transcripts. The resulting expression matrix was normalized via the LogNormalize method (scale factor = 10,000), and the top 2000 highly variable genes were identified using the variance-stabilizing transformation algorithm. Following multi-sample layer consolidation via the JoinLayers function, data scaling was applied to the variable genes. Principal Component Analysis (PCA) was then executed to capture the top 30 principal components. Finally, cells were partitioned into 26 discrete clusters using a shared nearest neighbor graph-based algorithm with a resolution of 0.5 and visualized via UMAP.

### 4.3. Cell Type Annotation and Tumor Microenvironment Analysis

Cell clusters were annotated based on canonical lineage-specific marker genes to resolve the cellular architecture of the gastric microenvironment. Specifically, *EPCAM* and *KRT8* were used to identify epithelial cells; *CD3D* for T cells; *CD14* for macrophages; *CD79A* for B cells; *MZB1* for plasma cells; *TPSAB1* for mast cells; *DCN* for fibroblasts; *VWF* for endothelial cells; *ACTA2* for smooth muscle cells; and *PLP1* for Schwann cells.

To reveal the dynamic remodeling of the gastric microenvironment and characterize the specific intercellular communication networks between epithelial cells and key stromal and immune compartments, including macrophages, T cells, and fibroblasts, cell–cell communication analysis was performed using CellChat (v2.0) [35]. To capture stage-specific variations, the microenvironmental cellular composition was stratified according to the pathological progression across CAG, IM, and GC stages. Stage-specific communication networks were constructed utilizing the CellChatDB.human database, with communication probabilities modeled via the triMean approximation method.

### 4.4. Pathway Enrichment Analysis

To decipher the biological functions and molecular pathways of the candidate genes, Gene Ontology (GO) and Kyoto Encyclopedia of Genes and Genomes (KEGG) pathway enrichment analyses were performed using the *clusterProfiler* package (version 4.1). For GO functional annotation, the genes were cross-referenced against the *org.Hs.eg.db* human genome database across three domains, including Biological Process (BP), Cellular Component (CC), and Molecular Function (MF), while the Homo sapiens database was utilized for KEGG mapping. Terms with both *p*.adjust < 0.05 and *q*.value < 0.05 were considered significantly enriched.

### 4.5. Pseudotime Trajectory Analysis of Gastric Epithelial Cells

To investigate the dynamic transcriptional changes and key regulators driving epithelial transformation during gastric carcinogenesis, pseudotime trajectory analysis was performed on the gastric epithelial cell subset using the Monocle2 (v2.22.0) package [36]. The temporal ordering was guided by the top 2000 highly variable genes identified. After calculating size factors and estimating dispersions under a negative binomial distribution, dimension reduction was executed using the DDRTree algorithm to map cells along a continuous developmental trajectory from normal epithelial states to malignant phenotypes. To further resolve expression dynamics along the trajectory, pseudotime-dependent differentially expressed genes were identified using the differentialGeneTest function. Genes exhibiting a q.value < 0.05 were defined as significantly trajectory-dependent, and were subsequently subjected to hierarchical clustering based on their kinetic profiles to dissect chronologically coordinated molecular shifts.

### 4.6. Structural Modeling of CagA–MARK2 Complex

The crystal structure of the CagA multimerization motif (CM) peptide (residues 948–961) bound to the MARK2 kinase domain (PDB ID: 3IEC) was utilized as the initial structural template. To investigate the functional impact of geographical polymorphisms, sequence variations between the Western and East Asian CagA lineages were identified through pairwise sequence alignment of genomic records retrieved from GenBank. Specifically, the genomic sequences of the prototype Western strain 26695 (United Kingdom) and the East Asian clinical isolate HLJ039 (China) were utilized as references for the Western and East Asian CagA models, respectively [37,38]. Within the CM region, the characteristic East Asian LRR-SAA motif from strain HLJ039 was introduced to replace the Western LKR-HDK motif from strain 26695. These targeted amino acid substitutions were performed using the mutate residues tool in Discovery Studio Visualizer. The resulting complex structures were subsequently prepared for MD simulations by adding missing hydrogen atoms and assigning standard amino acid protonation states corresponding to a physiological pH of 7.0.

### 4.7. Molecular Dynamics Simulation

All MD simulations were conducted using the AMBER 24 software package [39]. The Amber ff14SB force field was applied to describe the protein and peptide parameters. Each system was solvated in an octahedral box of TIP3P water molecules, with a minimum buffer distance of 10 Å between the solute and the box boundary. To neutralize the system and mimic physiological ionic strength, Na^+^ and Cl^−^ ions were added to a concentration of 0.15 M.

The simulation workflow proceeded through a meticulously optimized four-stage protocol. First, each system was subjected to 2000 cycles of initial energy minimization utilizing the steepest descent method followed by the conjugate gradient algorithm to eliminate spatial steric clashes. Subsequently, the systems were gradually heated from 0 K to a physiological temperature of 310 K over 20 ps under NVT conditions via a linear thermal ramp. To optimize solvent packing and resolve water density anomalies, a targeted short-term density equilibration was then performed under the NPT ensemble using the CPU-based pmemd engine to strictly stabilize the box density at approximately 1.0 g/cm^3^. Finally, unrestrained production MD simulations were executed for 100 ns using the GPU-accelerated pmemd.cuda engine.

Throughout the simulation, temperature was regulated at 310 K using Langevin dynamics, and pressure was maintained at 1 atm via the Monte Carlo barostat. Long-range electrostatic interactions were computed using the Particle Mesh Ewald (PME) method with a real-space cutoff of 10.0 Å. Covalent bonds involving hydrogen atoms were constrained using the SHAKE algorithm, allowing an integration time step of 2 fs. Trajectory stability was systematically assessed by evaluating the RMSD of the protein backbone.

### 4.8. Binding Free Energy Calculation (MM/GBSA) and Per-Residue Decomposition

To quantify the binding affinity between MARK2 and CagA isoforms, the binding free energy (Δ*G_bind_*) was calculated using the MM/GBSA method implemented in the MMPBSA.py module in AmberTools. Representative conformation snapshots were evenly extracted from the stable production trajectory for energetic evaluation. Prior to free energy calculation, explicit water molecules and neutralizing ions were stripped utilizing cpptraj to generate clean, dry topology files for the complex, receptor, and ligand systems, respectively. The total binding free energy (Δ*G_bind_*) was determined using the *igb* = 2 implicit solvation model and decomposed into the following energetic components:

Δ*G_bind_* = Δ*E_elec_* + Δ*E_vdw_* + Δ*G_GB_* + Δ*G_SA_* − *T*Δ*S* (1)

where Δ*E_elec_* and Δ*E_vdw_* represent the gas phase electrostatic and van der Waals interactions, respectively, while Δ*G_GB_* represents the polar solvation energy solved via the generalized Born equation, and Δ*G_SA_* represents the nonpolar solvation contribution estimated from the solvent-accessible surface area (SASA), and −*T*Δ*S* corresponds to the conformational entropy change. To align with our focus on individual molecular determinants, the −*T*Δ*S* term was not decomposed into per residue terms due to its inherent global nature. Instead, the final affinity estimation and energetic profiling relied exclusively on enthalpic decomposition to identify critical structural drivers, as conformational entropy typically cancels out when evaluating relative binding trends in highly homologous systems. Furthermore, to comprehensively map the structural determinants driving these lineage-specific polymorphism-mediated affinity shifts and to pinpoint how specific residue alterations between the two isoforms dictate the enhanced binding capacity, per-residue binding energy decomposition (*idecomp* = 1) was strictly restricted to the CagA multimerization motif (residues 948–961) to isolate individual thermodynamic hotspot amino acids.

## 5. Conclusions

In conclusion, this study provides an integrative analysis linking environmental pathogen genetic divergence, host microenvironmental toxic stress, and gastric oncogenesis. Our single-cell transcriptomic profiling delineates cell state transitions during *H. pylori-associated* progression, highlighting a distinct cytotoxic cascade where epithelial polarity alteration aligns with the disruption of neuroendocrine-like secretory and synaptic programs during late-stage transformation. Mechanistically, atomistic MD simulations and MM/GBSA thermodynamic analyses clarify the structural basis underlying this lineage-specific virulence. We show that the East Asian CagA peptide achieves an optimized thermodynamic balance, utilizing a localized hydrophobic interlocking mode to minimize polar desolvation penalties and engage the host MARK2 kinase domain with persistent structural occupancy, while maintaining peripheral dynamic flexibility. Collectively, by correlating interfacial dynamics with cell-state phenotypes, these findings significantly advance our mechanistic understanding of how this biological environmental carcinogen compromises epithelial safety barriers. Furthermore, our insights uncover potential lineage-specific interfacial hot-spots as promising targets for mitigating CagA-induced chronic cytotoxicity, offering new avenues for future therapeutic risk stratification and environmental health interventions.

## Data Availability

The single-cell transcriptomic datasets analyzed in this study are available in the NCBI Gene Expression Omnibus database under accession number GSE249874. The reference amino acid for the East Asian and Western *H. pylori* CagA strains were retrieved from the NCBI GenBank database.

## Supplementary Materials

The following supporting information can be downloaded at: https://www.mdpi.com/article/10.3390/molecules31173118/s1, Figure S1: Gene Ontology and KEGG pathway enrichment profiles of Cluster 3 genes. (A) Cellular Component terms. (B) Biological Process terms. (C) Molecular Function terms. (D) Bar plot illustrating the KEGG pathways; Figure S2: Ligand–receptor pairs mediating crosstalk between fibroblasts and epithelial cells; Figure S3: Ligand–receptor pairs mediating crosstalk between T cells and epithelial cells; Figure S4: Thermodynamic energy contribution analysis of Western and East Asian CagA lineages binding to MARK2; Figure S5: Contact occupancy analysis of key residue interactions in CagA–MARK2 complexes; Table S1: Pseudotime-dependent DEGs and co-expression modules along the evolutionary axis.

## Abbreviations

The following abbreviations are used in this manuscript:

*H. pylori*	*Helicobacter pylori*
CAG	Chronic atrophic gastritis
IM	Intestinal metaplasia
GC	Gastric cancer
CagA	Cytotoxin-associated gene A
VacA	Vacuolating cytotoxin A
scRNA-seq	Single-cell RNA sequencing
MD	Molecular dynamics
GEO	Gene Expression Omnibus
PCA	Principal Component Analysis
KEGG	Kyoto Encyclopedia of Genes and Genomes
GO	Gene Ontology
BP	Biological Process
CC	Cellular Component
MF	Molecular Function
CM	CagA multimerization motif
RMSD	Root Mean Square Deviation
SASA	Solvent-accessible surface area
DEGs	Differentially expressed genes
RMSF	Root mean square fluctuation
