# Supplementary material for: Lineage-Specific CagA Binding Mechanics and Microenvironmental Rewiring in East Asian Gastric Carcinogenesis
Source: Molecules. 2026 Sep 6;31(17):3118. doi: 10.3390/molecules31173118 (PMC13567578; doi:10.3390/molecules31173118)
Supplement: Supplementary file 1 [file molecules-31-03118-s001.zip › molecules-4479555-supplementary.pdf]

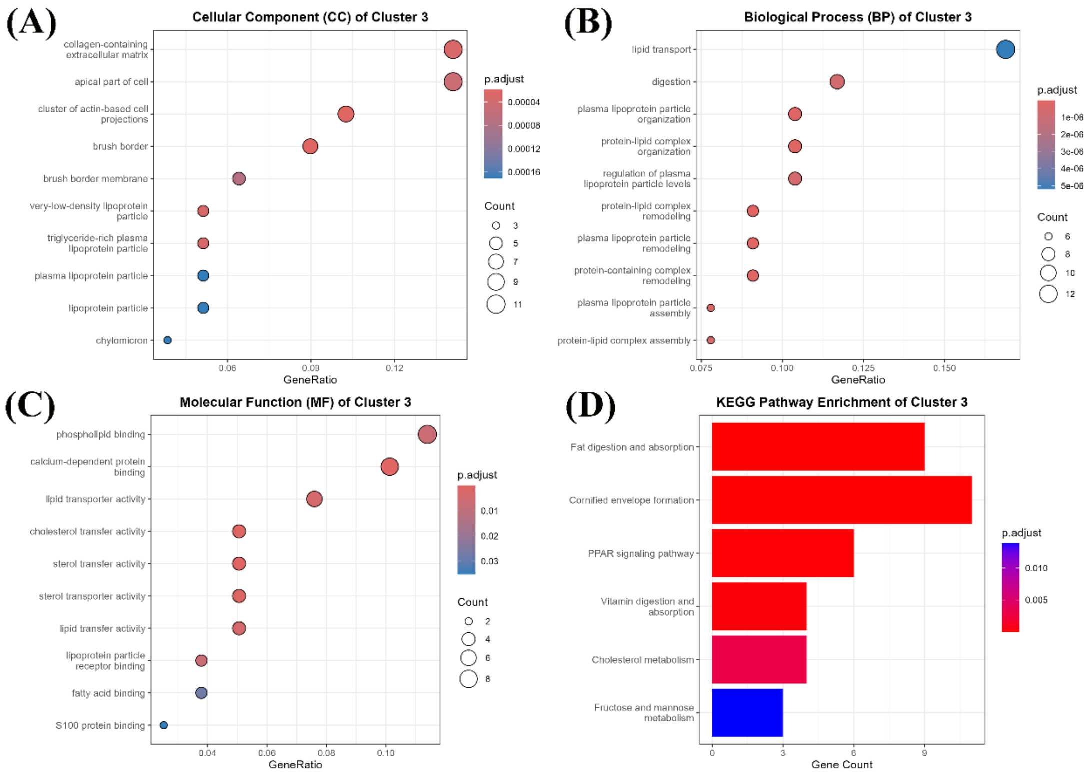

Figure S1. Gene Ontology and KEGG pathway enrichment profiles of Cluster 3 genes. (A) Cellular Component terms. (B) Biological Process terms. (C) Molecular Function terms. (D) Bar plot illustrating the KEGG pathways.

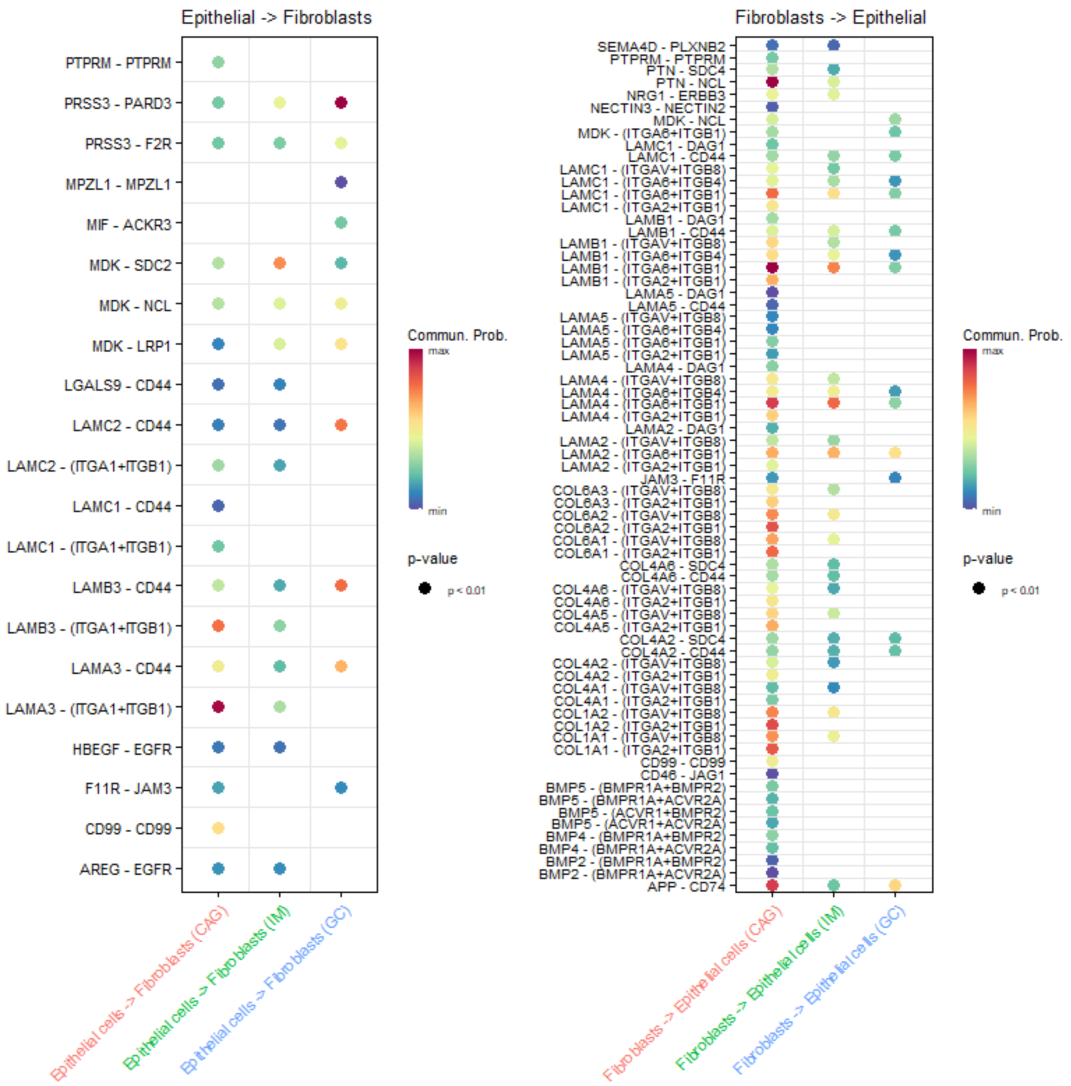

Figure S2. Ligand-receptor pairs mediating crosstalk between fibroblasts and epithelial cells.

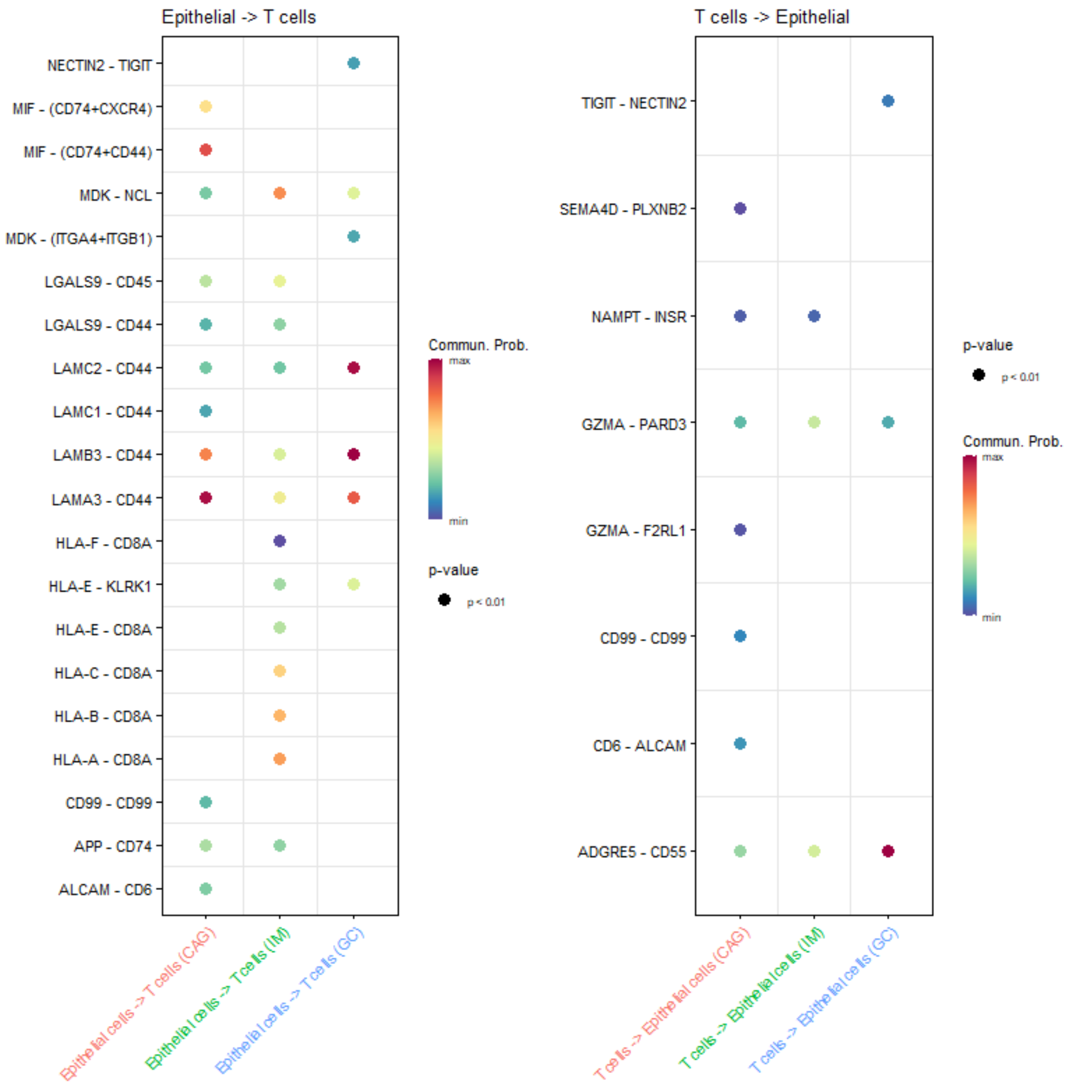

Figure S3. Ligand-receptor pairs mediating crosstalk between T cells and epithelial cells.

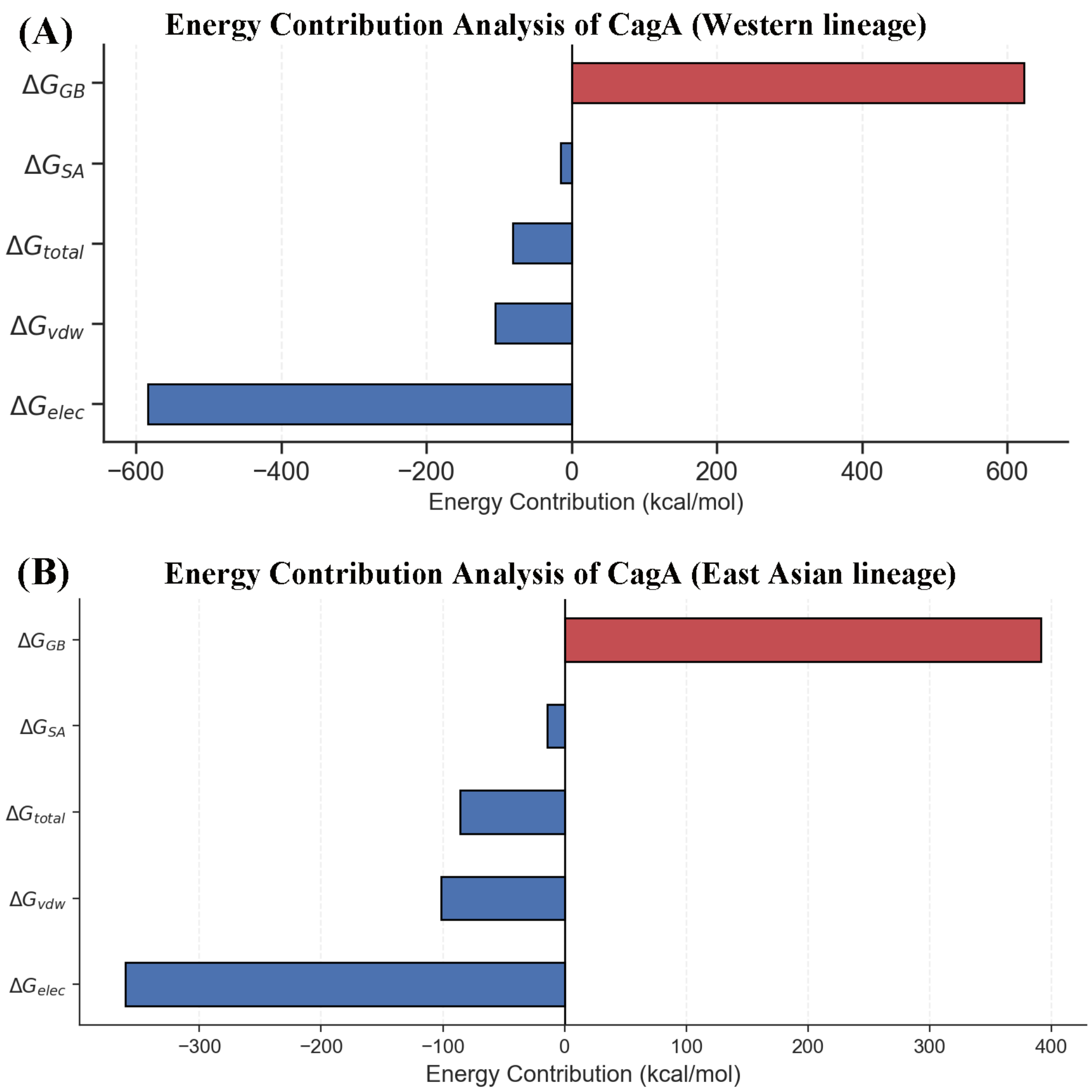

Figure S4. Thermodynamic energy contribution analysis of Western and East Asian CagA lineages binding to MARK2.

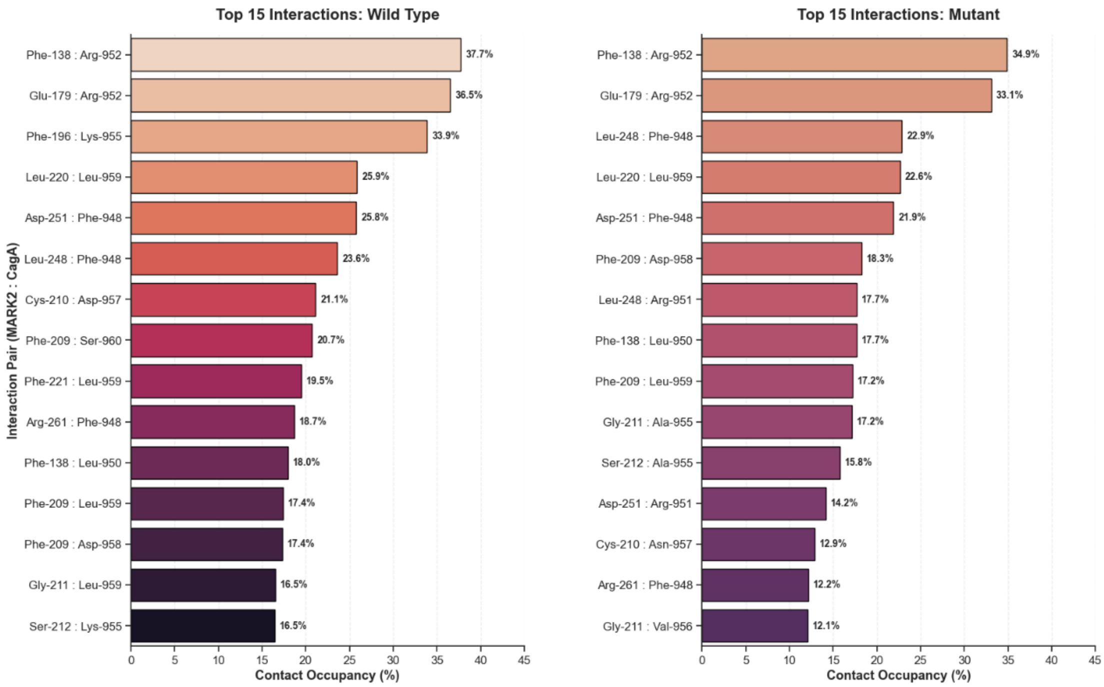

Figure S5. Contact occupancy analysis of key residue interactions in CagA–MARK2 complexes.

Table S1 Pseudotime-dependent DEGs and co-expression modules along the evolutionary axis.

Gene	Cluster	pvalue	qvalue	Gene	Cluster	pvalue	qvalue
SST	1	0	0	INSM1	1	6.23E-243	5.37E-242
MLN	1	0	0	NEGR1	1	7.97E-243	6.84E-242
GCG	1	0	0	AFF3	1	2.43E-242	2.07E-241
GHRL	1	0	0	PPFIA2	1	1.82E-241	1.55E-240
TTC26	1	0	0	SMOC1	1	3.87E-241	3.27E-240
TTR	1	0	0	ENSG00000227681	1	1.38E-240	1.16E-239
TRIM5	1	0	0	PLCXD3	1	2.89E-240	2.42E-239
CHGB	1	0	0	AK5	1	2.88E-239	2.40E-238
C6orf58	1	0	0	UNC79	1	2.43E-235	2.01E-234
TPH1	1	0	0	LY6H	1	8.28E-235	6.82E-234
LRP1B	1	0	0	DPYSL3	1	4.23E-234	3.47E-233
RALYL	1	0	0	PRUNE2	1	1.65E-232	1.35E-231
CHGA	1	0	0	TBC1D4	1	2.16E-232	1.76E-231
SHISAL2B	1	0	0	ISL1	1	1.12E-229	8.99E-229
GPC6	1	0	0	ADGRV1	1	3.13E-229	2.51E-228
SLC4A10	1	0	0	ARX	1	3.98E-229	3.17E-228
HS6ST3	1	0	0	RAB3B	1	5.33E-225	4.21E-224
KCNH7	1	0	0	KCTD8	1	5.41E-225	4.26E-224
GAST	1	0	0	TUSC3	1	5.73E-222	4.49E-221
PLD5	1	0	0	BMERB1	1	1.39E-220	1.09E-219
LINC00907	1	0	0	PDK3	1	3.93E-220	3.06E-219
KCNB2	1	0	0	UNC5D	1	9.53E-220	7.39E-219
DACH2	1	0	0	PAK3	1	2.58E-219	1.99E-218
NPAS3	1	0	0	DISC1FP1	1	4.31E-218	3.32E-217
LINC01811	1	0	0	HAP1	1	9.51E-218	7.28E-217
SCG2	1	0	0	LRRIQ1	1	8.48E-217	6.48E-216
GALNT13	1	0	0	CA8	1	1.52E-215	1.16E-214
SCG5	1	0	0	ITGB8	1	1.89E-214	1.43E-213
DTNA	1	0	0	ASCL1	1	3.10E-213	2.34E-212
LINGO2	1	0	0	MTUS2	1	1.53E-211	1.15E-210
PCSK2	1	0	0	EGR3	1	5.70E-210	4.25E-209
KCNMB2	1	0	0	BEX1	1	4.72E-209	3.49E-208
PCSK1N	1	0	0	NFASC	1	2.84E-208	2.09E-207
NLGN1	1	0	0	ETV1	1	3.28E-207	2.41E-206
CPE	1	0	0	EYS	1	6.51E-207	4.75E-206
MARK1	1	0	0	HMGCLL1	1	8.41E-206	6.12E-205
SNAP25	1	0	0	DIRC3-AS1	1	8.89E-205	6.44E-204
FSTL4	1	0	0	KCNJ3	1	3.53E-204	2.55E-203
DMD	1	0	0	RBP1	1	1.24E-202	8.91E-202

ANK2	1	0	0	CGA	1	9.18E-201	6.51E-200
CACNA2D1	1	0	0	SLC35F3	1	3.96E-199	2.80E-198
CACNB2	1	0	0	SLC26A7	1	1.44E-197	1.01E-196
HOMER1	1	0	0	C2CD4B	1	2.02E-197	1.41E-196
SLC38A11	1	0	0	ZC3HAV1	1	1.49E-195	1.03E-194
PAM	1	0	0	MARCHF1	1	1.39E-194	9.56E-194
CACNA1A	1	0	0	SPOCK3	1	1.73E-192	1.19E-191
SYNPR	1	0	0	FMN2	1	4.42E-192	3.02E-191
FGF14	1	0	0	PTPRN	1	4.90E-191	3.34E-190
NRXN1	1	0	0	KLHL32	1	6.09E-191	4.13E-190
PTPRT	1	0	0	NEUROD1	1	9.35E-191	6.32E-190
SLC8A1	1	0	0	GIP	1	4.34E-189	2.90E-188
RIMS2	1	0	0	PDE10A	1	4.73E-188	3.15E-187
CRYBA2	1	0	0	LIPF	2	0	0
TMEM132D	1	0	0	IGKC	2	0	0
AGBL4	1	0	0	TFF2	2	0	0
ADCY2	1	0	0	PGC	2	0	0
ERO1B	1	0	0	MUC5AC	2	0	0
MAGI2	1	0	0	AGR2	2	0	0
RORA	1	0	0	PGA5	2	3.89E-301	4.03E-300
RGS17	1	0	0	LAMA3	2	1.24E-265	1.15E-264
RGS7	1	0	0	LCN2	2	7.83E-258	7.11E-257
DDC	1	0	0	RASEF	2	2.31E-255	2.07E-254
CASR	1	0	0	SPINK1	2	1.08E-244	9.39E-244
MIR7-3HG	1	0	0	IGLC3	2	4.62E-230	3.72E-229
DACH1	1	0	0	IGLC2	2	8.02E-202	5.73E-201
KCNMA1	1	0	0	IGHA1	2	7.19E-197	5.01E-196
SOX5	1	0	0	IGHA2	3	0	0
TUBA1A	1	0	0	PLA2G2A	3	0	0
RMST	1	0	0	FABP1	3	0	0
SOGA3	1	0	0	APOA4	3	0	0
DNAJC12	1	0	0	APOC3	3	0	0
ENSG00000225689	1	0	0	GKN1	3	0	0
PCSK1	1	0	0	RBP2	3	0	0
CDHR3	1	0	0	APOA1	3	0	0
CACNA1C	1	0	0	S100A4	3	0	0
CADPS	1	0	0	CEACAM5	3	0	0
ST18	1	0	0	TM4SF20	3	0	0
PDZRN3	1	0	0	PI3	3	0	0
KCTD12	1	0	0	CEACAM6	3	0	0
TSHZ2	1	0	0	KRT20	3	0	0
RFX6	1	0	0	FABP2	3	0	0
STXBP5L	1	0	0	GKN2	3	0	0

MAPK10	1	0	0	DMBT1	3	0	0
MS4A8	1	0	0	MUCL3	3	0	0
RTN1	1	0	0	TMPRSS15	3	0	0
KCNH8	1	0	0	PRAP1	3	0	0
TOX	1	0	0	PHGR1	3	0	0
SCG3	1	0	0	ANPEP	3	0	0
SDK1	1	0	0	SLC26A3	3	0	0
NBEA	1	0	0	MUC17	3	0	0
RIMBP2	1	0	0	ALDOB	3	0	0
NOL4	1	0	0	APOB	3	0	0
CDK14	1	0	0	MTTP	3	0	0
SAMD5	1	0	0	ISG15	3	0	0
DRAIC	1	0	0	IL32	3	0	0
TMOD1	1	0	0	C11orf86	3	0	0
HEPACAM2	1	0	0	TFF1	3	0	0
RAPGEF4	1	0	0	GDA	3	0	0
KCNJ6	1	0	0	HKDC1	3	0	0
OXR1	1	0	0	SLC15A1	3	0	0
SPAG16	1	0	0	CES2	3	0	0
ARHGEF38	1	0	0	S100A11	3	0	0
SCGN	1	0	0	HHLA2	3	0	0
MAP1B	1	0	0	S100A6	3	0	0
PCLO	1	0	0	ADIRF	3	0	0
LINC01876	1	0	0	TSPAN8	3	0	0
TMEM178B	1	0	0	LGALS3	3	0	0
LIMCH1	1	0	0	S100A16	3	0	0
UBR1	1	0	0	SDCBP2	3	0	0
RFX3	1	0	0	AGPAT2	3	0	0
KCNMB2-AS1	1	0	0	AKR1B10	3	0	0
FEV	1	0	0	S100A10	3	0	0
CADM1	1	0	0	AGR3	3	0	0
PBX3	1	0	0	S100A14	3	0	0
SORBS2	1	0	0	PCSK5	3	0	0
PROX1	1	0	0	KRT19	3	0	0
SETBP1	1	0	0	CRIP1	3	0	0
MAN1A1	1	0	0	TRIM31	3	0	0
DLG2	1	2.96E-323	3.31E-322	DGAT1	3	0	0
DPP6	1	1.88E-321	2.08E-320	SFN	3	0	0
NCAM1	1	3.16E-321	3.48E-320	C19orf33	3	0	0
SLC7A2	1	1.24E-319	1.35E-318	ABTB2	3	1.89E-306	2.00E-305
RAB3C	1	5.34E-316	5.81E-315	CLDN7	3	2.51E-303	2.63E-302
GNG4	1	3.42E-315	3.70E-314	ACSL5	3	1.52E-295	1.54E-294
CHN2	1	5.51E-313	5.92E-312	HSD17B2	3	2.03E-290	2.01E-289

SCGB2A1	1	0.00E+00	5.39E-308	CDHR5	3	8.37E-278	8.01E-277
DOCK4	1	9.61E-308	1.02E-306	KRT17	3	2.82E-276	2.69E-275
PAX6	1	1.08E-304	1.13E-303	PCK1	3	2.00E-272	1.88E-271
MX2	1	2.26E-301	2.35E-300	EMP1	3	3.32E-270	3.10E-269
SNED1	1	7.41E-299	7.64E-298	ANXA1	3	5.71E-261	5.21E-260
SAMD3	1	8.01E-298	8.21E-297	PRSS3	3	1.50E-255	1.35E-254
LINC01013	1	2.34E-297	2.39E-296	BNIP5	3	2.31E-253	2.06E-252
UNC80	1	1.76E-296	1.79E-295	PLAUR	3	5.18E-249	4.59E-248
ITPR1	1	4.49E-292	4.51E-291	FER1L6	3	2.63E-246	2.31E-245
CHRNA7	1	6.44E-291	6.44E-290	MUC3A	3	9.20E-245	8.04E-244
KCNT2	1	1.96E-290	1.95E-289	CD9	3	3.81E-243	3.30E-242
ZNF385D	1	7.37E-287	7.26E-286	HPGD	3	3.51E-241	2.97E-240
PEG10	1	1.43E-283	1.41E-282	MALL	3	3.60E-238	2.99E-237
SSTR2	1	2.35E-283	2.30E-282	LINC01610	3	2.64E-232	2.14E-231
MAOB	1	1.68E-282	1.64E-281	RHOC	3	8.23E-229	6.53E-228
RUNX1T1	1	4.17E-282	4.03E-281	IL1RN	3	2.31E-211	1.73E-210
ASTN2	1	4.75E-282	4.57E-281	PLAAT2	3	2.57E-209	1.91E-208
ENSG00000233928	1	1.35E-273	1.28E-272	C3orf85	3	2.61E-207	1.92E-206
RCAN2	1	1.51E-270	1.42E-269	LGALS4	3	9.47E-204	6.81E-203
PRICKLE2	1	4.77E-268	4.44E-267	LAMC2	3	4.25E-201	3.03E-200
DLGAP1	1	5.74E-265	5.29E-264	TYMP	3	1.44E-197	1.01E-196
ERC2	1	8.58E-264	7.87E-263	CDHR2	3	7.80E-197	5.42E-196
DSCAML1	1	1.90E-257	1.72E-256	ACE2	3	1.59E-195	1.10E-194
LINC00261	1	6.63E-256	5.97E-255	SPP1	3	1.17E-189	7.86E-189
MYH10	1	2.04E-246	1.80E-245	GPA33	3	3.65E-189	2.45E-188
